# A Probabilistic Approach to Assessing and Predicting the Failure of Notched Components

**DOI:** 10.3390/ma12244053

**Published:** 2019-12-05

**Authors:** Miguel Muñiz-Calvente, Lucas Venta-Viñuela, Adrián Álvarez-Vázquez, Pelayo Fernández Fernández, María Jesús Lamela Rey, Alfonso Fernández Canteli

**Affiliations:** Department of Construction and Manufacturing Engineering, University of Oviedo, Campus de Viesques, 33203 Gijón, Spain; Uo252984@uniovi.es (L.V.-V.); alvarezvadrian@uniovi.es (A.Á.-V.); fernandezpelayo@uniovi.es (P.F.F.); mjesuslr@uniovi.es (M.J.L.R.); afc@uniovi.es (A.F.C.)

**Keywords:** Notch effect, generalised local model, probability of failure

## Abstract

This work presents a probabilistic model to evaluate the strength results obtained from an experimental characterisation program on notched components. The generalised local method (GLM) is applied to the derivation of the primary failure cumulative distribution function (PFCDF) as a material property (i.e., independent of the test type, load conditions and specimen geometry selected for the experimental campaign), which guarantees transferability in component design. To illustrate the applicability of the GLM methodology, an experimental program is performed using specimens of EPOLAM 2025 epoxy resin. Three different samples, each with a specific notch geometry, are tested. As a first scenario, a single assessment of each sample is obtained and the PFCDFs are used to perform cross predictions of failure. Some discrepancies are noticeable among the experimental results and cross-failure predictions, although they are within the expected margins. A possible reason for the disagreement can be assigned to the inherent statistical variability of the results and the limited number of tests per each sample. As a second scenario, a joint assessment of the three samples is performed, from which a unique PFCDF is provided, according to the GLM. In the latter case, a more reliable assessment of the experimental results from the geometry conditions is achieved, the suitability of the selected driving force is verified, and the transferability of the present material characterisation is confirmed.

## 1. Introduction

The presence of notches in structural components exemplifies one of the most common causes of fracture and fatigue failure in real structures under service. As a result, the current practical design is confronted with the effect of different complex notched-type structural details, such as joints or holes, which act as stress concentrators, thus influencing the fracture and fatigue design assessment according to the directives of the structural integrity concept.

Because of the practical relevance of the above problem, different deterministic models have been developed in the last decades that aim to solve the notch effect due to stress concentrations. Among them, some efforts have been devoted to calculating the influence of the stress distribution based on the stress intensity factor concept within a large variety of notch types and geometries, such as Peterson’s well-known works [1,2], summarised in reference [3].

On the other side, the model based on the theory of critical distances (TCD) proposed by Taylor [4,5] has worldwide recognition. As a whole, that model constitutes a group of methodologies, namely point, line, surface and volume methods, in terms of the definition of the characteristic material parameter, denoted the critical distance (L). In the case of the point method, a fracture is expected to occur when the stress reaches the inherent strength (usually higher than the ultimate tensile strength of the material) at a distance of L/2 from the defect tip. In turn, the line method establishes that a fracture occurs when the average stress along a distance equal to 2L overcomes the inherent strength; the concept is similarly extended to the surface and volume methods.

More recently, other models have emphasised the influence of external effects (e.g., temperature) on the fracture characterisation of notched-type elements, such as the notch master curve method [6] derived by applying the former master curve method to the failure analysis in the ductile-to-brittle transition in metals [7,8]. Finally, other approaches, based on the original TCD, have been presented during the few last years with the aim of including different effects, such as plasticity or constraints. Nevertheless, the vast majority of the above models only represent deterministic approaches.

The significant and non-negligible scatter associated with fracture results has incited the development of probabilistic models. Some probabilistic models, similarly to certain deterministic models, have been set up to take into account external effects, such as temperature, as well as the notch influence as a combined effect acting on the apparent fracture toughness of metallic materials [9,10]. However, such models cannot guarantee the suitability of the selected parameter (driving force), to which the failure criterion is referred. Furthermore, they ignore the consideration of a scale effect arising from the non-uniform stress distribution present in the structural component. As a consequence, neither the reciprocal conversion of the fracture characterisation results among different kind of tests nor the transferability from lab results to real component designs is guaranteed.

Accordingly, this paper presents a novel probabilistic methodology to achieve an independent and unbiased evaluation of the experimental results gathered from diverse fracture characterisation programs. The main purposes of the proposed methodology are fourfold: (a) to univocally obtain a mathematical relation between the value of the selected reference or failure parameter (i.e., the driving force) and the probability of failure, independently of the geometrical notch characteristics (e.g., radius and size); (b) to propose a procedure for validating the suitability of the selected failure criterion; (c) to ensure the transferability of the results among experimental campaigns characterised by different notch geometries and sizes, as well as the transferability from the laboratory test results to the design of real components and (d) to promote the joint evaluation of different experimental campaigns to enhance the assessment reliability.

An EPOLAM 2025 epoxy resin was characterised with the aim of illustrating the applicability of the proposed methodology. Such a resin is commonly used in the aeronautical sector. In particular, three experimental samples of EPOLAM 2025, provided with different notch geometries, were tested and characterised in this work.

The paper is organised as follows: After a general introduction and motivation of the investigation, Section 2 presents the experimental program. Thereafter, the methodology, based on the generalised local model [11] is introduced and explained step by step in Section 3. It comprises a single evaluation of each experimental subprogram and cross-failure prediction, as well as a joint evaluation of the whole experimental program and validation of the selected reference parameter (driving force). In Section 4 the results obtained are discussed and, finally, in Section 5, the main conclusions of the work are drawn.

## 2. Introduction to the Experimental Program Used to Illustrate the Methodology

### 2.1. Material

Polymeric materials play an overwhelming role in the design and manufacturing of advanced structural components, as, for example, the polymeric composites in the aeronautical industry. The high competitiveness arising among the industries of this sector compels engineers to develop materials with increased strength and reduced weight. Consequently, the characterisation of those new materials is essential to ensure their applicability and usefulness. In this sense, suitable probabilistic methodologies allow the inherent scatter of the failure results to be taken into account in the test data assessment and in the subsequent design of the component.

In this work, the epoxy resin EPOLAM 2025 was selected as a suitable material to be characterised because of its extensive use on aeronautics. To maintain the same mechanical properties of the material throughout the experimental campaign, the traceability of all samples was ensured. The epoxy resin was supplied by AXSON Technologies (Barcelona, Spain), the manufacturing process of the resin plates was carried out by the National Institute of Aerospace Technology (Madrid, Spain) and the specimens were machined by Prodintec (Gijón, Spain).

The mechanical properties of the EPOLAM 2025 resin were obtained from a uniaxial tensile test showing a Young’s Modulus, E = 2200 MPa, and a Poisson coefficient, μ = 0.36.

### 2.2. Geometry, Test Plan and Procedure

**Figure 1:**
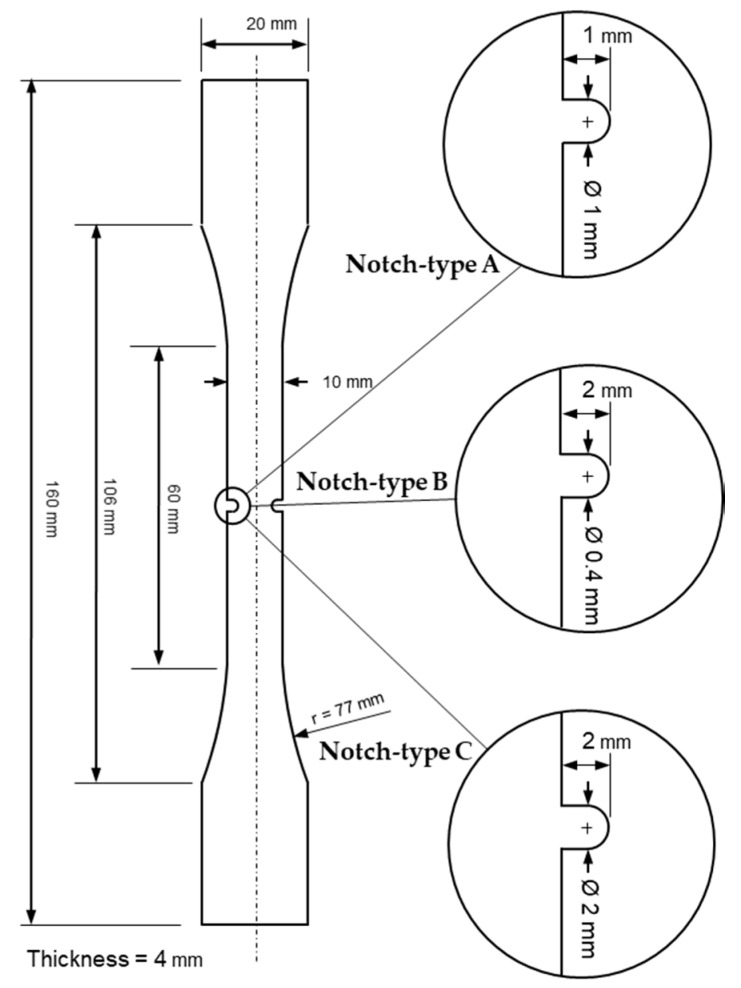
Geometry and notch details of the three samples tested in the experimental program.

To illustrate the proposed methodology for failure prediction in polymeric materials, an experimental campaign was carried out in which dog bone specimens, each exhibiting a particular notch type, were used. Figure 1 shows the geometry and main dimensions of the three different notched specimen types. The experimental program consists of eight A-type specimens, six B-type specimens and five C-type specimens.

All tests were performed at 16 ± 2°C in an MTS Series 642 testing machine (MTS Sensor Technologie GmbH & Co. KG, Luedenscheid, Germany) equipped with a 5 kN load cell by applying a displacement control at a speed of 5 mm/min.

### 2.3. Results

**Figure 2:**
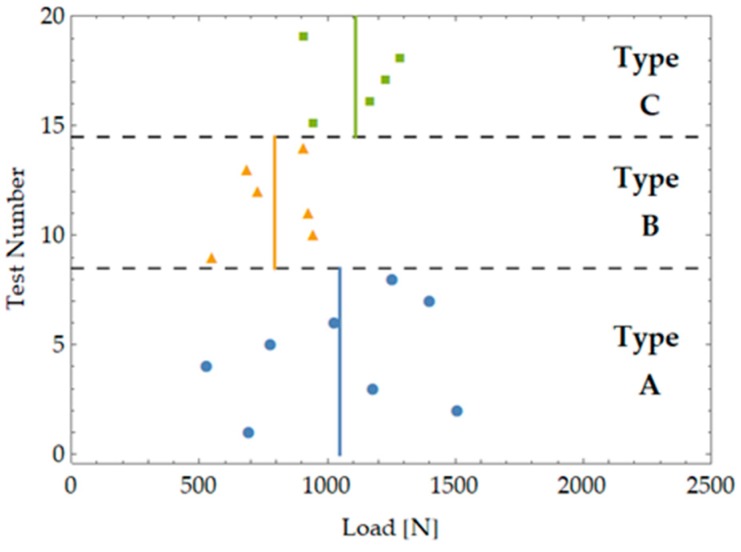
Test results for the three samples tested representing three different notch types.

Figure 2 and Table A1 show the maximum load values at failure for the performed tests along with the average value for each sample.

## 3. Proposed Methodology

**Figure 3:**
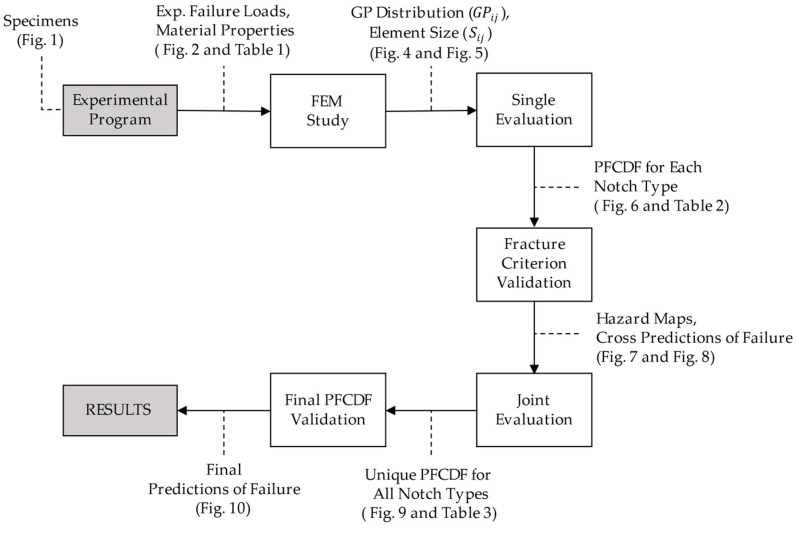
Schematic representation of the proposed methodology.

This section describes the steps required to derive and validate the primary failure cumulative distribution function (PFCDF) according to the generalised local model (GLM), as proposed in reference [11]. The PFCDF represents a material property, thus it should be an objective and unique probabilistic description of the characterisation, irrespective of notch geometry and size of the samples tested. The flowchart in Figure 3 summarises the methodology proposed with the main inputs and outputs at each step involved in the procedure. The first step consisted of performing an experimental program that allowed us to know the critical loads related to the failure of the specimens tested. Afterwards, a finite element model (FEM) was used to obtain the distribution of the generalised parameter ($GP_{ij}$) for each specimen subjected to the critical loads registered. Following, the $GP_{ij}$ and the size of each element in the model ($S_{ij}$) were used to obtain the PFCDF for each notch type by applying the GLM. With the aim of validating the suitability of the failure criterion selected, once the PFCDF for each notch type was known, different cross predictions of failure among the different notch types under study were performed and compared with the experimental results observed in the laboratory. If the cross predictions agree with the experimental results, it is possible to perform a joint evaluation of all samples to obtain a unique PFCDF with a higher reliability, which can be used to predict the failure for any type of notch. The following subsections describe the main steps of the methodology.

### 3.1. Finite Element Model

**Figure 4:**
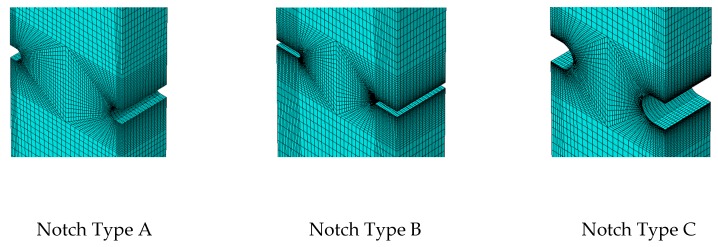
Finite element meshes.

To determine the critical stress conditions at failure, the progressive loading process at each test was simulated using the commercial FEM software ABAQUS 6.12 (Dassault Systèmes Simulia Corp, Johnston, RI, USA) [12]. In the case of EPOLAM, an epoxy resin which exhibits a clear brittle behaviour at failure, the maximum principal stress has been found to be the adequate generalized parameter (GP) to which failure of the material is referred to. In any case, other failure criteria could be envisaged as possible candidates in a more complete study, where the methodology applied is the same as that proposed here. According to the above, the FE analysis focused on the calculation of the local values of that parameter during the application of the remote load (i.e., for different and incremental load steps). Figure 4 shows a view of the mesh used, which consisted of around 100,000 linear four-node brick elements with reduced integration (C3D8R). With the aim of reproducing the tests conditions prescribed by ASTM D638 (2004) [13] for this kind of test, the lower part of the specimen was fixed whereas the final load acting at the test end was applied at the upper part of the specimen. The validation of the FEM was confirmed by comparing the reaction forces and the local displacements at the upper part of the specimens of the numerical model with those results obtained experimentally. In all the cases, a highly satisfactory fitting was observed.

**Figure 5:**
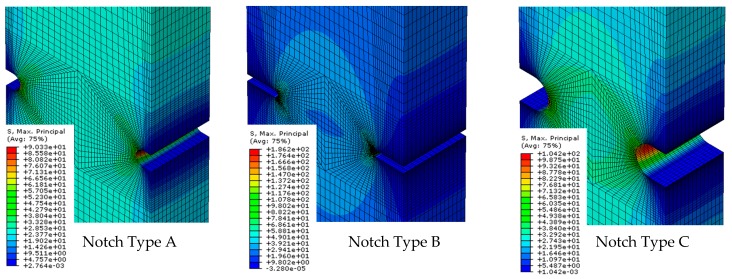
Example of principal maximum stresses distribution.

Once all simulations were accomplished, the postprocessing tool Abaqus2Matlab [14] was used to automatically export the following variables for each test (j) from Abaqus to Matlab (See Figure 5):

- The maximum principal stress reached at each element (i) for the failure load: $GP_{ij}$
- The size of each element (i): $S_{ij}$

### 3.2. Single Evaluation of Each Notch Type

To evaluate the experimental results obtained, the GLM developed by the authors [11] was used to derive the PFCDF for each sample. The GLM aimed to obtain the PFCDF as a material property independent of the type of test performed, or in other words, irrespective of the loading conditions and geometry and size of the specimen used in the test. According to reference [15], the probabilistic model, as related to a strength problem, should be based on the Weibull distribution function for minima, as given by Equation (1):

(1) $$P_{fail}=1-exp\left[-{\left(\frac{GP-\lambda }{\delta }\right)}^{\beta }\right];\;GP\;>\;\lambda ,$$

where GP represents the selected driving force and *β*, *λ* and *δ* are the shape, location and scale parameters of the Weibull distribution, respectively. The PFCDF provides an unequivocal relation between the probability of failure and the GP value when the latter is applied uniformly on a specific size, denoted as reference size $\left(S_{ref}\right)$, associated with the scale parameter.

**Figure 6:**
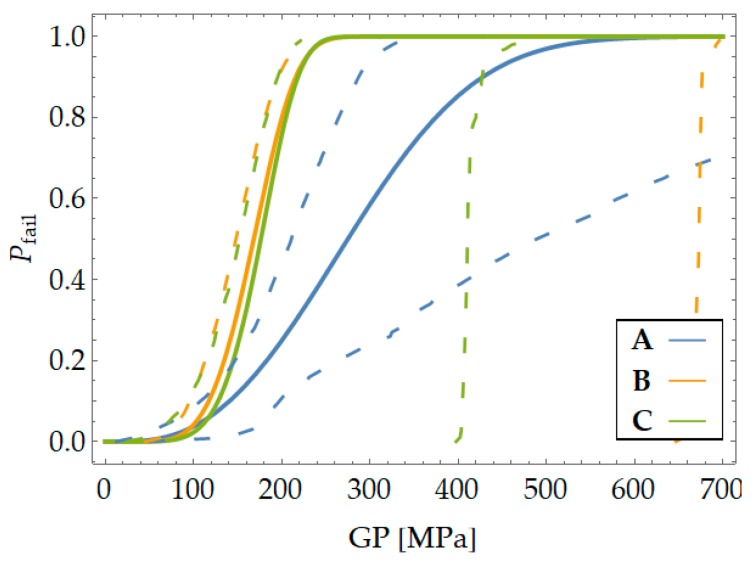
Failure cumulative distribution functions (CDFs) obtained from each notch type ($S_{ref}=1\;{cm}^{3}$).

Figure 6 and Table 1 show the three PFCDFs calculated by applying the GLM to the experimental results from the previous test program. When the results from the three samples were evaluated in a separated way, they did not appear to fully confirm the previous expectations concerning the uniqueness of the three, making PFCDFs a material property that should not depend on geometry or test conditions. It is possible that the large scatter observed in the experimental results, along with the limited number of tests within each sample under the same test conditions, led to the broad confidence intervals in the PFCDF definition. Regardless, the confidence intervals of the respective PFCDFs overlap in some way, thus providing a possible justification for the observed disagreement. The validity of those PFCDFs is discussed in the following section.

### 3.3. Validation of the Primary Failure Cumulative Distribution Functions

To ensure the reliability of the PFCDFs and the transferability of the results among the three experimental programs carried out, the PFCDF obtained for each notch type was used to derive the joint cumulative distribution function prediction failure for all the samples as a whole. To do so, local and global probabilities of failure were obtained, taking into account the particular stress distributions associated to each notch type and their influence on the scale effect.

First of all, the size of any element ($S_{ij}$) on a finite element mesh can be different from the reference size ($S_{ref}$) defined in the previous section. For that reason, it was necessary to enrich Equation (1) by including the scale effect (See Equation (2)):

(2) $$P_{{fail}_{ij}}=1-exp\left[-\frac{S_{ij}}{S_{ref}}{\left(\frac{GP_{ij}-\lambda }{\delta }\right)}^{\beta }\right];\;GP_{ij}>\;\lambda$$

**Figure 7:**
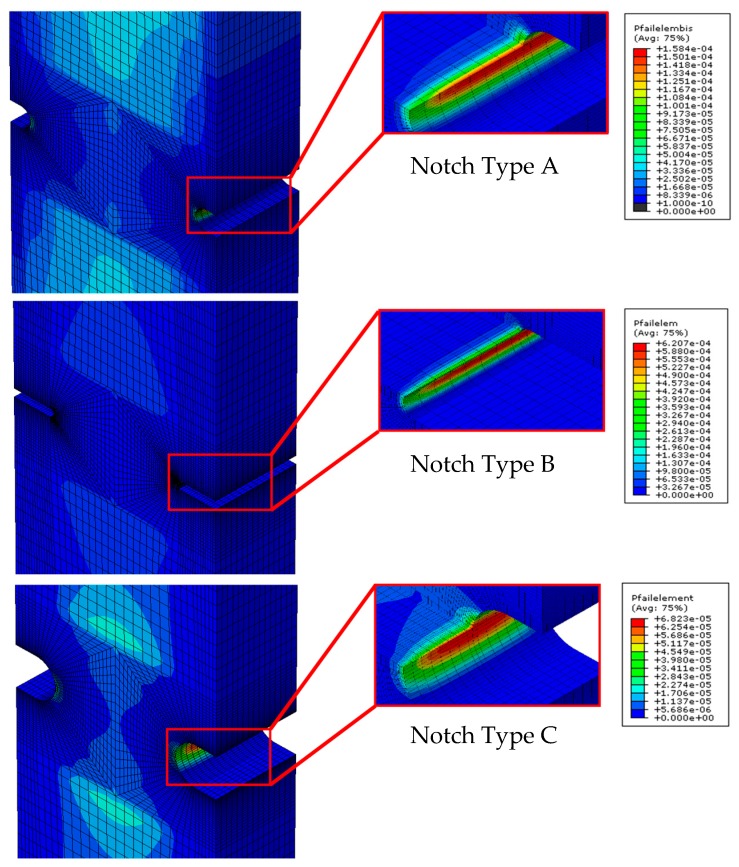
Example hazard maps: distribution of the local probability of failure for notch type A, B and C (remote applied load 1000 N).

Equation (2) provides the probability of failure for a finite element associated to a size ($S_{ij}$) subject to $GP_{ij}.$ To proceed to the prediction of the local probabilities of failure for each notch type sample, the remote load was gradually incremented from zero to a value that guaranteed a global (i.e., for the specimen as a whole) probability of failure near 99.9%. For example, Figure 7 shows the local probabilities of failure (hazard maps [16]) for the three notch type samples associated to a remote load of 1000 N. Those predictions were made using Equation (2) particularised to the PFCDF associated with Notch Type B. 

Once the hazard maps were obtained, the evolution of the global probability of failure associated to the remote load applied could be obtained by applying the weakest link principle defined by Equation (3):

(3) $$P_{fail_{j}}=1-\prod_{i=1}^{n}\left(1-P_{fail_{ij}}\right)=1-\prod_{i=1}^{n}exp\left[-\frac{S_{i}}{S_{ref}}{\left(\frac{GP_{ij}-\lambda }{\delta }\right)}^{\beta }\right];\;GP_{ij}>\;\lambda .$$

**Figure 8:**
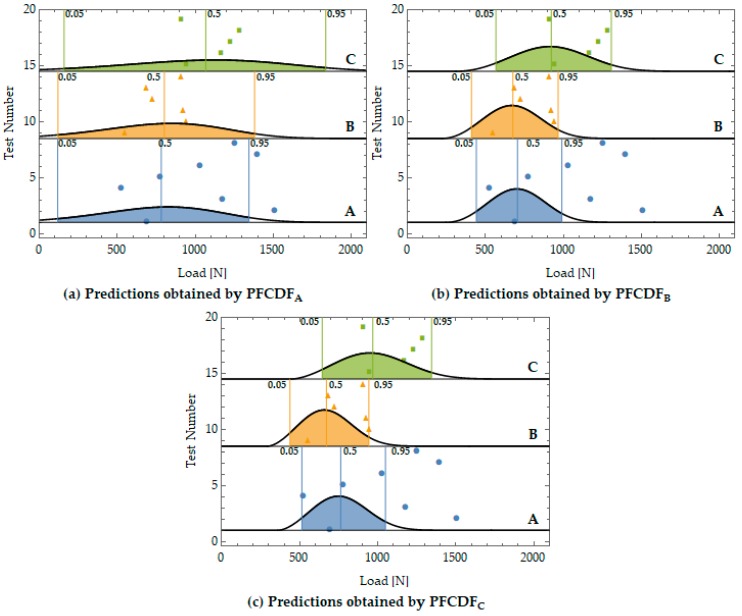
Comparison between the experimental results and the predictions of global probabilities of failure (*p* = 0.05, 0.5 and 0.95) estimated from each PFCDF: Type A (**a**), Type B (**b**) and Type C (**c**)**.**

As can be seen in Equation (3), the global probability of failure depends on the values of the Weibull parameters (λ,δ and β). Thus, different predictions can be made depending on the PFCDF adopted to perform the prediction (See Table 1). Taking into account that the Weibull parameters should be material properties, it is possible to predict the failures of a certain notch type based on the PFCDF associated to another notch type, which means that cross predictions of failure between different notch types would be performed. Figure 8a shows the predictions of failure (0.05, 0.5 and 0.95 global probabilities of failure) obtained from the PFCDF associated to Notch Type A, Figure 8b from Notch Type B and Figure 8c from Notch Type C. These graphs represent the cross predictions of failure because the experimental results and conclusions associated to a particular notch type (i.e., A) were used to predict the failure for the other notch types (i.e., B and C).

As can be seen in Figure 8, the cross predictions and the experimental results agreed relatively well because the vast majority of points were on the band *p* = 0.05−0.95 predicted. Nevertheless, some experimental results were found not to be properly distributed around the predicted *p* = 0.50 probability of failure or to fall outside the expected probability of failure range, *p* = 0.05−0.95. To prove that this solution is possible and that the possible deviations can be associated to the scatter of the experimental results and the reduced size of the experimental campaign, the Anderson-Darling statistical hypothesis test was used. This hypothesis test was applied to each pair of estimated failure cumulative distribution functions and the experimental data samples. The results proved that, in all the cases, the experimental results could pertain to the distribution function associated with the prediction of failure for a significance level α = 0.10.

### 3.4. Joint Evaluation of All Notch Types

From the point of view of reliability, two possible alternatives in the planification of an experimental program could be recommended as suitable. In the first, a unique experimental program comprising all the tests is carried out, seemingly providing a unique PFCDF with enhanced reliability because of the higher number of tests performed under identical features. In the second, *n* experimental subprograms (i.e., more than one) of different features are carried out to probe the validity of the GP to transfer results from one type of experiment to another, thus leading to *n* single PFCDFs with moderate reliability because of the smaller number of specimens tested within each of the subprograms. Nevertheless, by resorting to the GLM, it is feasible to derive a unique PFCDF from the *n* single PFCDFs, which ensures more reliably of the material characterization because it encompasses the randomness of the participating test subprograms. Furthermore, the GLM allows the failure criterion selected to be checked and possibly validated by applying cross predictions, as mentioned above.

**Figure 9:**
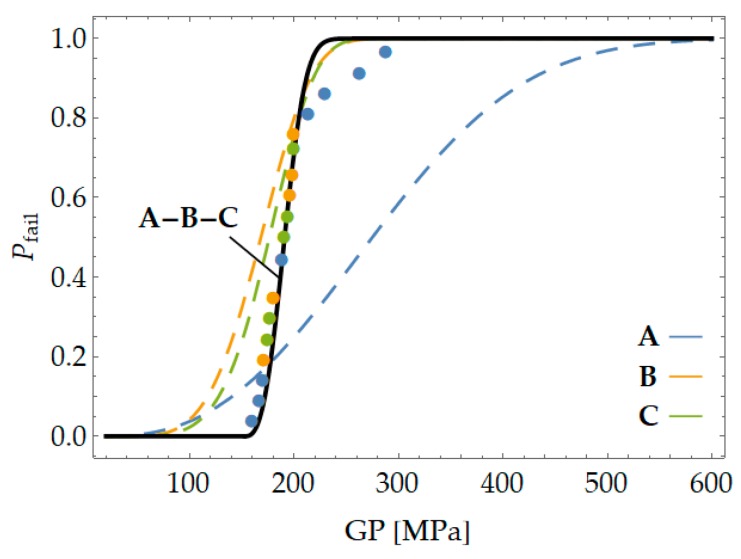
PFCDF obtained by the joint assessment ($S_{ref}=1\;{cm}^{3}$).

Accordingly, the next step was a procedure to obtain a unique PFCDF from several PFCDFs as derived from all the experimental subprograms. To do so, all results were referred to the same reference size [17,18], and finally a unique PFCDF with narrower confidence intervals than the previous ones was obtained (See Figure 9 and Table 2).

In this case, the suitability of the GP is confirmed in Figure 9 because the results for each sample are randomly and homogenously distributed along the PFCDF, or, in other words, all along the 0–1 probability range.

### 3.5. Validation of the Primary Failure Cumulative Distribution Function

**Figure 10:**
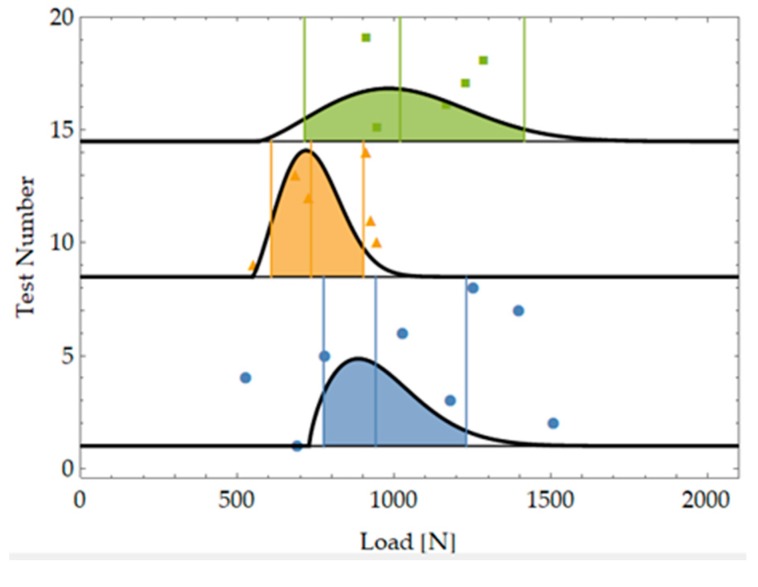
Comparison between 0.05; 0.5 and 0.95 global probabilities of failure estimated by the joint PFCDF and the real experimental results.

The joint PFCDF obtained in the previous step was used to derive the global failure cumulative distribution function predicted for each notch type sample following the same procedure described in Section 2.3. A comparison between the real and predicted experimental failures provided information about the quality of the final PFCDF derived. As can be seen in Figure 10, the predictions agreed well with the experimental results in all cases, as confirmed by the statistical hypothesis test that had already been performed.

## 4. Discussion

In view of the results obtained in this work, the PFCDF can be corroborated as a material property. Based on the PFCDF, the applicability of the methodology to failure prediction presented was confirmed by cross derivations of the PFCDFs obtained from different test conditions.

The joint evaluation, implying all the tests from the different notch type samples, improved the reliability associated with the prediction of failure. In this way, an optimal relation could be found between the probability of failure and the GP for a given reference size.

Although different PFCDFs were obtained from the particular notch types considered in the experimental program, only a final average PFCDF arose as a representative for the material independently of the three different types of tests performed. This proves that the methodology presented in this paper is applicable to the characterisation of the same material using to any other type of notched components.

The methodology presented in this paper to achieve the assessment of failure prediction of notched components was confirmed by its application to the evaluation of experimental results from tests using notched specimens made of EPOLAM 2025 epoxy resin, mainly due to the potential interest of this characterisation for different purposes in aeronautical designs. Its application can be extended and recommended to any other type of brittle material.

## 5. Conclusions

The following principal conclusions can be drawn from this paper:

- Based on the GLM, a unique PFCDF was derived from the experimental results as a material failure property (i.e., independent of the test type, load conditions and specimen geometry and size selected for the experimental campaign). The PFCDF guarantees transferability in a component design.
- The PFCDF can be applied to predict failure for and from different notch types (cross predictions), and its suitability was confirmed by assessing the results from an experimental program performed on EPOLAM 2025 epoxy resin specimens with three different notch types.
- The high scatter inherent to the experimental strength results of EPOLAM 2025 attests to the necessity of applying probabilistic models to their assessment if transferability and safe designs of components are intended.
- The GLM allowed the test data of this material to be jointly evaluated as pertaining to a unique sample, irrespective of the diversity of the notch geometries and an unequal or even scarce number of results per sample.

## Figures and Tables

**Table 1:** Weibull parameters of each primary failure cumulative distribution function (PFCDF) ($S_{ref}=1\;{cm}^{3}$).

Test Type	λ	β	δ
A	15.27	2.59	299.01
B	31.62	4.02	149.70
C	21.83	5.00	166.29

**Table 2:** Weibull parameters of joint PFCDF ($S_{ref}=1\;{cm}^{3}$).

Test Type	λ	β	δ
A-B-C	152.72	2.77	43.68

## Appendix A

**Table A1:** Experimental results obtained for the three samples tested

Test Number	Test Type	Failure Load (*N*)
1	A	694.94
2	A	1510.32
3	A	1182.13
4	A	529.92
5	A	780.55
6	A	1031.98
7	A	1401.93
8	A	1256.07
9	B	554.39
10	B	948.04
11	B	928.92
12	B	730.49
13	B	689.41
14	B	912.04
15	C	948.64
16	C	1171.09
17	C	1230.23
18	C	1288.51
19	C	911.936
